# SARS-COV-2 mutations in North Rift, Kenya

**DOI:** 10.1371/journal.pone.0325133

**Published:** 2025-06-06

**Authors:** Elius Mbogori, Kelvin Thiongo, Harrison Yunying Deng, Caroline Wangui Gikunyu, Winfrida Cheriro, Stanslaus Musyoki, Richard Biegon, Damaris Matoke-Muhia, Kirtika Patel, Binhua Liang, Elijah Songok

**Affiliations:** 1 Laboratory Department, Moi Teaching and Referral Hospital, Eldoret, Kenya; 2 School of Medicine, Moi University, Eldoret, Kenya; 3 Center for Biotechnology Research and Development, Kenya Medical Research Institute, Nairobi, Kenya; 4 Faculty of Arts and Science, University of Toronto, Toronto, Ontario, Canada; 5 Department of Medical Laboratory Sciences, South Eastern Kenya University, Kitui, Kenya; 6 Vaccines and Therapeutics, Medical and Scientific Affairs, National Microbiology Laboratory Branch, Public Health Agency of Canada, Winnipeg, Canada; 7 Department of Biochemistry and Medical Genetics, Max Rady College of Medicine, University of Manitoba, Winnipeg, Canada; 8 Center for Virus Research, Kenya Medical Research Institute, Nairobi, Kenya; Emory University, UNITED STATES OF AMERICA

## Abstract

The rise of new SARS-CoV-2 mutations brought challenges and progress in the global fight against COVID-19. Mutations in spike and accessory genes affect transmission, vaccine efficacy, treatments, testing, and public health strategies. Monitoring emerging variants is crucial to halt re-emergency of the virus and spread. 44 nasopharyngeal/oropharyngeal swabs from Kenyan patients were sequenced with the Illumina platform. Galaxy’s bioinformatic tools were used for genomic analysis. SARS-CoV-2 genome classification was done using PANGOLIN and mutation annotation with the COVID-19 Annotator tool. From this study, 5 clades of SARS-CoV-2 were identified of whom 38 (86%) were BA.1.1; 2 (5%) were BA.1.1.1; 1 (2%) was BA.1; 1 (2%) was BA.1.14 and 2 (5%) were AY.46. Symptomatic patients were 16 out of 18 males and 22 out of 26 females. Out of these, symptomatic patients, BA.1.1 was found in 14 males and 18 females. In these clades we found 53 significant mutations of which 42 were non-synonymous, 10 synonymous, 7 deletions, 4 insertions and 2 extragenic. Out of the 42 non-synonymous mutations, 7 were exclusively found in symptomatic patients. Two new mutations, S:R214R, and NSP2:A555A, were also found and were dominant in symptomatic patients. These findings add to the understanding of the SARS-CoV-2 virus future evolution in the region.

## 1. Introduction

The emergence of novel mutations of SARS-CoV-2 introduced new challenges and breakthroughs in the global fight against the COVID-19 pandemic. These mutations in the viral genome, have impacted various aspects of the pandemic response [1,2]. Notably, mutations in the spike gene and other accessory genes have been identified, affecting transmission dynamics, vaccine efficacy, disease severity, therapeutic interventions, diagnostic testing, and public health measures [3,4]. Within the spike gene, several mutations such as R203K, G204R, N501Y, E484K, L452R, and E484Q have been identified, which have the potential to increase viral replication, fitness, and immune evasion [3,4].

Additionally, the D614G mutation in the spike protein has been found to influence the pathogenesis of SARS-CoV-2 by enhancing spike protein trafficking to lysosomes, resulting in decreased spike expression on the cell surface [5]. Epistasis, the interaction between mutations, has also been studied extensively in the context of SARS-CoV-2. Research has shown that mutations in the receptor binding domain (RBD) of the spike protein can confer resistance to neutralizing antibodies, indicating complex interactions between mutations and antibody recognition [6]. Studies have identified sites under strong and weak epistatic constraints, highlighting their roles in viral replication and pathogenicity [7]. Furthermore, deep mutational scans have revealed epistatic shifts in the effects of mutations, suggesting that certain substitutions shape subsequent evolutionary changes [8]. Bernando et al. (2022) also found that substitutions in the RBD can cause epistatic shifts, influencing the virus’s affinity for the ACE2 receptor [9].

In Kenya, the pandemic has been characterized by multiple waves, each with unique features. Initial waves occurred before the emergence of variants of concern (VoCs) and had lower attack rates [10]. Subsequent waves, driven by Alpha, Delta, and Omicron VoCs, exhibited higher attack rates [10]. Various studies conducted across different regions in Kenya have shown fluctuations in the prevalence of SARS-CoV-2 over different time periods, highlighting the region important for the virus evolving and dynamic nature. For instance, a nationwide seroprevalence study indicated an overall prevalence of about 9.4% among blood donors, with variations across different demographics and regions [11]. In Nairobi, the rates reached as high as 34.7% [12]. This disparity can be attributed to several factors, including differences in population density, mobility patterns, and public health interventions. Very little has however been published on North Rift Kenya.

The Omicron variant, first identified in South Africa, gained worldwide recognition due to its numerous mutations, particularly in the spike protein’s receptor-binding domain [13]. The occurrence and rise of new strains of the SARS-CoV-2 virus have drawn global attention and caused concern. These changes can affect how easily the pathogen spreads, how severe the disease is, or its ability to escape immune system responses.

Every new mutation brings more uncertainty about what it could mean for the pandemic. Worries include the possibility of faster community spread and potential reduced efficacy of existing vaccines. The emergence of new mutations among general populations can be a cause of confusion and anxiety. These further fuels fear and panic, especially in places already severely hit by the virus. This emphasis on novel strains mirrors international anxiety to understand and tackle COVID-19 urgently. To keep the virus from spreading, there has to be constant monitoring alongside research as well as cooperation between countries.

## 2. Methods

### 2.1. Samples collection

Swab samples and were obtained from the oropharynx/nasopharynx of patients attending Moi Teaching and Referral Hospital in Eldoret, North Rift Kenya in December 2021 subsequent to obtaining ethics approval from Moi University/Moi Teaching and Referral Hospital Institutional Research Ethics committee (Ref. IREC/036/2021 and ELD/MTRH/R&P/10/2/V.2/2010). All study participants provided written informed consent. Participants’ clinical details were obtained by the resident clinician at the specimen collection site. The samples were then preserved in viral transport medium (VTM) and transported to the laboratory within a period of 6 hours, adhering strictly to the cold chain, and subsequently stored at −20°C. Using the DaAn Gene Detection kit for 2019-nCoV (DaAnGene, China) on the Rotor Gene Q real-time PCR machine, reverse transcription-polymerase chain reaction (RT-PCR) analysis was performed. All samples yielding positive results for SARS-CoV-2 with a cycle threshold (CT) value less than 30 were earmarked for sequencing.

### 2.2. Next-generation sequencing

Prior to sequencing, samples underwent preparation procedures. Initially, reverse transcription (RT) was carried out using the SuperScript™ IV Reverse Transcriptase kit from Illumina, enabling the conversion of RNA into complementary DNA (cDNA). Subsequent to this, cDNA underwent amplification via polymerase chain reaction (PCR) targeting specific SARS-CoV-2 genes, facilitated by the NEBNext® Ultra™ II DNA Library Prep Kit. During library preparation, adapters tailored for Illumina sequencing were affixed to the amplified fragments. Cleanup procedures were then conducted utilizing the Agencourt AMPure XP system, followed by size selection to eliminate contaminants. Quantification of library concentration was performed using Qubit to ensure an optimal loading concentration. Finally, sequencing was executed using the MiSeq sequencing platform (Illumina, California, USA), adhering strictly to the manufacturer’s stipulated protocols [14].

### 2.3. Genomic analyses and variants assessments

After to sequencing, bioinformatics analyses were performed using the Galaxy platform. Initial quality assessments of sequencing data were conducted using FastQC. This was followed by sequence trimming using the trim sequence tool and alignment to the reference SARS-CoV-2 genome (NCBI accession number MN908947.2) [15] using bowtie2 [16]. Duplicate sequences were removed via the Mark duplicates tool. Variant calling was done using FreeBayes [17], followed by variant filtering utilizing VCF filter to ensure criteria met for read depth (DP > 30), allele frequency (AF > 0.2), and variant quality (QUAL >30). The consensus tool was then employed to generate a fasta file combining variant information.

Further categorization of SARS-CoV-2 genome sequences into genetic lineages was achieved utilizing the Phylogenetic Assignment of Named Global Outbreak LiNeages (PANGOLIN). Annotation of sequences was carried out using the COVID-19 Annotation tool (http://giorgilab.unibo.it/coronannotator/) [18].

To elucidate novel mutations, sequences from previous mutations were retrieved from GISAID database. These mutations were annotated utilizing the COVID-19 Annotation tool (http://giorgilab.unibo.it/coronannotator/) [18] for comparison with mutations identified in the study.

### 2.4. Data availability

The sequences obtained were published in the Global Initiative on Sharing All Influenza Data (GISAID) database.

## 3. Results

### 3.1. Sequencing data

The sequencing information from the set of 44 SARS-CoV-2 sequenced samples had an average of base coverage of 95%. Each base on average had a coverage depth of 282.73, with a maximum coverage depth reaching up to 1031.16. The sequences were deposited in GISAID and published with the accession numbers EPI_ISL_19004000 to EPI_ISL_19004016 and EPI_ISL_19004981 to EPI_ISL_19005007. All the sequences are listed in the S1 Table

### 3.2. SARS-CoV-2 variants of concern

**Fig 1:**
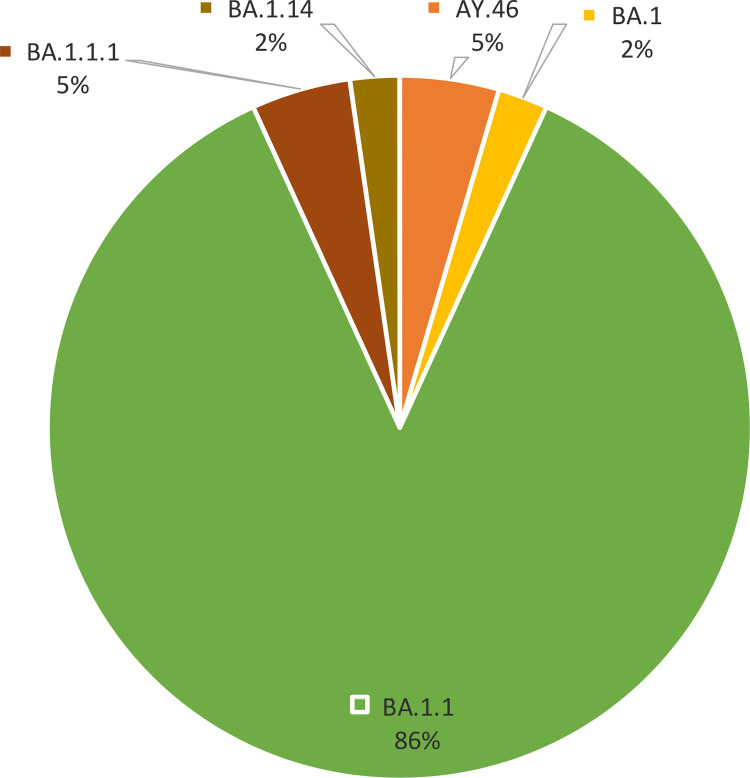
Clade frequency: The clade naming is based on PANGO system. BA.1 represents the original Omicron variant. Each subsequent number denotes a sub-variant of its preceding number. AY.46 is from the Delta variant*.*

The sequencing outcomes of SARS-CoV-2 encompassed a cohort of 44 samples. There were two variants of concern seen: forty two samples (95%) had Omicron variant while two samples (5%) had delta variant. Within the Omicron variant, a genetic diversity was observed, with four discernible clades denoted as BA.1, BA.1.1, BA.1.1.1, and BA.1.14. The BA.1.1 clade, domineered constituting a substantial majority at 86.4% of the total sequenced samples (Fig 1).

### 3.3. Variant distribution by gender among symptomatic and asymptomatic patients

Among the symptomatic patients, there were 16 (42%) males and 22 (58%) females. For symptomatic males, 14 (88%) cases had BA.1.1 variant. There is one case (6%) each for the BA.1 and AY.46 variants. No symptomatic males are reported for the BA.1.1.1 and BA.1.14 variants. For symptomatic females, 18 (82%) cases were due to the BA.1.1 variant. There were 2 (10%) cases of the BA.1.1.1 variant and one (5%) case each for the BA.1.14 and AY.46 variants. There were no symptomatic female cases for the BA.1 variant. Among the asymptomatic patients, there were 2 (33%) males and 4 (67%) females. All asymptomatic males and females were infected with the BA.1.1 variant. There were no asymptomatic cases for the BA.1.1.1, BA.1, BA.1.14, and AY.46 variants in either gender (Table 1).

**Table 1:** Distribution of the clades in relation to gender and symptoms.

Clade	Symptoms	Gender		
		Male	Female	Total
**BA.1.1**	Symptomatic	14	18	**32**
	Asymptomatic	2	4	**6**
**BA.1.1.1**	Symptomatic	0	2	**2**
	Asymptomatic	0	0	**0**
**BA.1**	Symptomatic	1	0	**1**
	Asymptomatic	0	0	**0**
**BA.1.14**	Symptomatic	0	1	**1**
	Asymptomatic	0	0	**0**
**AY.46**	Symptomatic	1	1	**2**
	Asymptomatic	0	0	**0**
**Total**	**Symptomatic**	**16**	**22**	**38**
	**Asymptomatic**	**2**	**4**	**6**
	**All**	**18**	**26**	**44**

### 3.4. Frequency of mutations in the population

A total of 65 mutations were seen in various samples at varying frequencies. The “5’UTR” region showed a variant at position 241 with a frequency of 31.8%. In the Non structural protein (NSP) 2, a mutation A555A at position 2470 is present in 18.2% of subjects. Multiple mutations were observed in the NSP3 protein, including F106F, I388L, A889A, S1265, and A1892T with frequencies ranging from 11.4% to 97.7% as shown in the S2 Table.

For the NSP4 protein, the T492I mutation at position 10029 was found in 97.7% of subjects. NSP6 showed variants like L105 and I189V, each with a frequency of 95.5%. NSP10 and NSP12b also displayed high-frequency mutations such as V57V and P314L, with the latter being present in all subjects (100%). NSP12b also includes N591N at a lower frequency of 11.4%. At NSP13, G170S mutation was observed in 63.6% of subjects. The NSP14 protein included the I42V mutation, present in 95.5% of subjects. For the Spike (S) protein, multiple mutations such as A67, T95I, I210, N211K, and others were observed, with frequencies ranging from 6.8% to 100%. Notably, the D614G variant in the Spike protein was found in all subjects (100%).

Other significant mutations included G339D, R346K, and S371L, with frequencies of 84.1%, 81.8%, and 22.7%, respectively. The ORF3a protein had a T64T mutation in 95.5% of subjects. Mutations in the Envelope (E) and Membrane (M) proteins, like T9I and Q19E, showed frequencies of 88.6% and 95.5%, respectively. The ORF6 and ORF7b regions included variants like M19M and L17L with frequencies of 95.5% and 61.4%, respectively.

**Fig 2:**
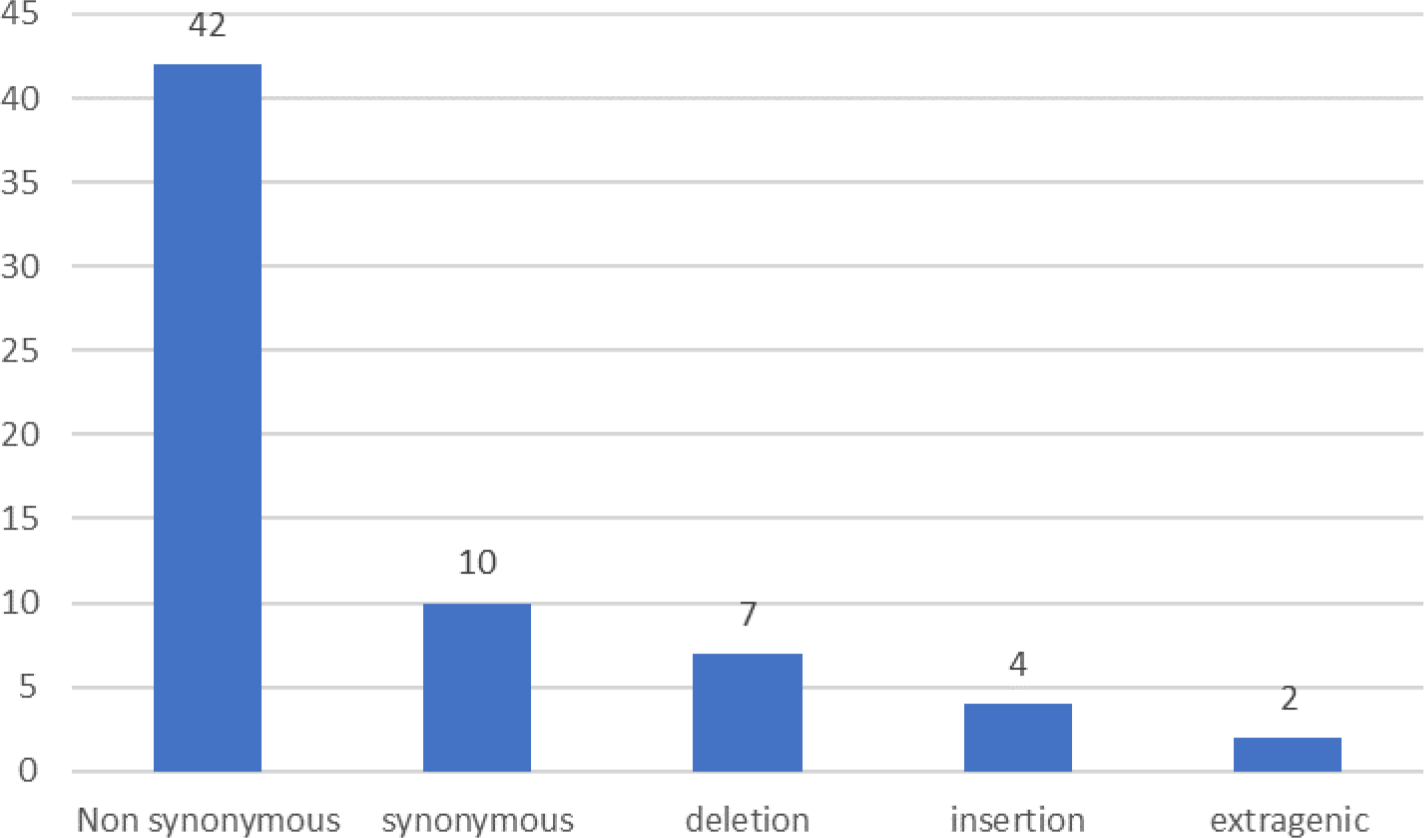
Distribution of various mutations in respect to their type.

Out of these 65 mutations that were seen in the study, 42 were non-synonymous, 10 were synonymous, 7 were deletions, 4 were insertions, and 2 were extragenic mutations in the **3’UTR** and **5’UTR** regions as shown in Fig 2.

Out of the 42 non- synonymous mutations, 7 mutations: NSP3:I388L; S:E484A; S:G446S; S:G496S; S:N211K; S:N440K; and S:N501Y were exclusively found in symptomatic patients. All the four insertions: S:I210; S:R214; S:S214; and S:V213, were found in symptomatic patients. Similarly, one synonymous mutation; S:R214R out of the 10 and one extragenic mutation: 5’UTR:241 out of the 2 were exclusively found in symptomatic patients. All the other mutations were seen in both symptomatic and asymptomatic patients.

### 3.5. Emerging mutations

Mutation data from previous waves in Kenya were analyzed alongside the mutations from this study. Two new mutations were noted: S:R214R accounting for 19 (43.2%) cases within the study and NSP2:A555A, is identified in 8 (18.2%) cases in the study group. All the patients who had S:R214R showed symptoms while 7 out of 8 patients with NSP2:A555A were symptomatic as shown in Table 2.

**Table 2:** New mutations.

Name	Cases in study	Symptomatic
S:R214R	19 (43.2%)	19 (100%)
NSP2:A555A	8 (18.2%)	7 (88%)

## 4. Discussion

From the 44 SARS-CoV-2 sequenced samples in this study Omicron was found to have dominance over other strains which compares to a study conducted in Mexico City, where Cedro-Tanda *et al*. (2022) reported that the Omicron variant significantly contributed to the increase in COVID-19 cases during the same period [19]. This is because when compared to prior variants, Omicron has demonstrated lower antibody neutralization, higher infectivity, and reduced vaccine effectiveness [20] Within Omicron, four sub-lineages were observed, with BA.1.1 accounting for 86.4%. This genetic diversity and the selective advantage of BA.1.1 are likely due to enhanced transmissibility and immune evasion as seen in other studies [20–22].

The study analyzed clinical and genomic data, where distinct patterns in the distribution of SARS-CoV-2 variants among symptomatic and asymptomatic patients across different gender groups were seen. Among symptomatic COVID-19 cases, females outnumbered males. The BA.1.1 variant was predominant in both groups, accounting for 88% of symptomatic males and 82% of symptomatic females. This suggests that the BA.1.1 variant may play a significant role in driving symptomatic infections, potentially due to its enhanced transmissibility and immune evasion capabilities. The data also reveal differences in the representation of other Omicron sub-lineages between genders. Symptomatic males showed less diversity, with only BA.1 and AY.46 each accounting for 6% of cases. In contrast, symptomatic females exhibited a more diverse variant profile, with BA.1.1.1, BA.1.14, and AY.46 also represented. The BA.1 variant was absent in symptomatic females, and no BA.1.1.1 or BA.1.14 cases were reported in symptomatic males. These findings suggest that there may be gender-specific differences in the susceptibility or immune response to different Omicron sub-lineages, potentially contributing to the observed disparities in variant distribution among symptomatic patients. This is in contrast with a previous study that reported no gender-based differences in immune responses and disease severity in COVID-19 [23].

A total 65 specific mutations occurring at frequencies ranging from 6.8% to 100% were identified across various structural and non-structural proteins in the study with most mutations occurring on spike protein. Non-structural proteins with mutations include NSP2, NSP3, NSP4, NSP6, NSP12b and NSP14. The role of each NSP has been studied and documented such as NSP3, which is responsible for viral replication and immune evasion. Each of these mutations influence the protein activity, potentially altering the virus’s ability to replicate and evade host defenses [24]. The impact of specific mutations in spike protein found in this study have been documented in previous studies. Mutations S:G446S, S:E484A, S:G496S, S:N440K, and S:N501Y in the spike protein of SARS-CoV-2 significantly contribute to the virus’s enhanced infectivity and immune evasion. Each mutation plays a crucial role. For instance S:G446S and S:E484A are particularly impactful in the context of the Omicron variant, altering antibody responses and the spike protein’s structure. S:G496S and S:N440K facilitate immune escape and modulate the spike protein’s functionality, while S:N501Y increases binding affinity to the ACE2 receptor and expands the virus’s host range. Together, these mutations underline the importance of monitoring SARS-CoV-2’s evolution to inform treatment and vaccination strategies. More research is however required to understand the importance of S:N211K and NSP3:I388L in the infectivity and pathogenicity of SARS-CoV-2

The most abundant new mutation in the region is S: R214R, a synonymous mutation, which account for 43.2% and of cases within the study. Previously, within the S1 subunit of the spike protein where this mutation lies, was an insertion S:R214 but progressively the mutation is changing to S:R214R. There is little information available for this change to a synonymous mutation. Synonymous mutations in SARS-CoV-2 have however been found to be functionally and evolutionarily significant. In the past, synonymous mutations were viewed as phenotypically silent but recent studies indicate they can affect viral fitness with potential functional associations [25,26]. These types of mutations may alter RNA secondary structure leading to a higher probability of base pairing [27]. Moreover, there is a different frequency and regularity of mutations in various SARS-CoV-2 genes, where some genes have more non-synonymous mutations than others [28]. In this study, all patients that had this mutation were symptomatic which calls for more research to find out the importance of this mutation.

## 5. Conclusion

The emergence of novel mutations and variants of SARS-CoV-2 has posed significant challenges in the global battle against the COVID-19 pandemic. The study found BA.1.1 clade as the most prevalent in the region. Other clades identified include BA.1, BA1.1.1, BA.1.14 and AY.46. Gender-based differences in variant distribution patterns among symptomatic and asymptomatic cases was observed which calls for further investigation to unravel the underlying biological, immunological, and sociological factors that may contribute to these disparities.

A total of 65 mutations both synonymous and non-synonymous were detected in the symptomatic and asymptomatic patients. The non-synonymous mutations G446S, E484A, G496S, N440K, and N501Y in the spike protein of SARS-CoV-2 seen exclusively in symptomatic patients are significant determinants of the virus’s infectivity, immune evasion, and resistance to neutralizing antibodies. Each mutation contributes uniquely to the virus’s ability to spread and evade immune responses.

Two new mutations, S:R214R and NSP2:A555A, were found. Although they are synonymous mutations, they were predominantly found in symptomatic patients. This necessitates further research in a larger scale to determine their significance in pathogenesis and immune evasion of SARS-CoV-2.

The findings from this study emphasize the need for continuous genomic surveillance and the integration of clinical and genomic data to elucidate the complex interplay between SARS-CoV-2 variants, host factors, and disease outcomes. By continuously monitoring the genetic landscape of the virus and understanding the implications of novel mutations, we can better adapt our public health strategies to control the spread of COVID-19 and mitigate its impact on global health and society.

## Supporting information

S1 Table Sequencing data and Clade for each genome. (DOCX)

S2 Table Mutation frequency for each mutated position in percentage. (DOC)

S1 Fig Evolutionary relationships SARS-CoV-2 genomes in North Rift Kenya. (PDF)
