# Supplementary material for: SARS-COV-2 mutations in North Rift, Kenya
Source: PLoS One. 2025 Jun 6;20(6):e0325133. doi: 10.1371/journal.pone.0325133 (PMC12143566; doi:10.1371/journal.pone.0325133)
Supplement: S1 Table — (DOCX) [file pone.0325133.s001.docx]

# Supplementary Table S1:

S1 Table: Sequencing data and Clade for each genome

| Sequence Name | Lineage | Bases with coverage | Average coverage depth | Maximum coverage depth |
| --- | --- | --- | --- | --- |
| hCoV-19/Kenya/SME008_MTRH_S13/2021 | AY.46 | 97.8 | 265.6 | 1000 |
| hCoV-19/Kenya/SME026_MTRH_S37/2021 | AY.46 | 93.6 | 36.5 | 142 |
| hCoV-19/Kenya/SME028a_MTRH_S7/2021 | BA.1.1 | 82.6 | 28.7 | 247 |
| hCoV-19/Kenya/SME029_MTRH_S49/2021 | BA.1.1 | 99.1 | 386.4 | 1222 |
| hCoV-19/Kenya/SME030_MTRH_S61/2021 | BA.1.1 | 91.4 | 74.5 | 414 |
| hCoV-19/Kenya/SME036_MTRH_S85/2021 | BA.1.1 | 98.4 | 514.8 | 1510 |
| hCoV-19/Kenya/SME037_MTRH_S2/2021 | BA.1.1 | 99.78 | 427.7 | 1.175 |
| hCoV-19/Kenya/SME038_MTRH_S14/2021 | BA.1.1 | 97.9 | 167.4 | 609 |
| hCoV-19/Kenya/SME039_MTRH_S26/2021 | BA.1.1 | 86.4 | 32.7 | 177 |
| hCoV-19/Kenya/SME041_MTRH_S38/2021 | BA.1.1.1 | 99.8 | 524.3 | 1572 |
| hCoV-19/Kenya/SME042_MTRH_S50/2021 | BA.1.1 | 87.9 | 41.9 | 239 |
| hCoV-19/Kenya/SME043_MTRH_S62/2021 | BA.1.1 | 97.8 | 196.2 | 684 |
| hCoV-19/Kenya/SME045_MTRH_S74/2021 | BA.1.1 | 99.2 | 489.8 | 1519 |
| hCoV-19/Kenya/SME047_MTRH_S86/2021 | BA.1.1 | 97.4 | 431.7 | 1325 |
| hCoV-19/Kenya/SME048_MTRH_S3/2021 | BA.1.1 | 96.25 | 71.23 | 290 |
| hCoV-19/Kenya/SME066_MTRH_S15/2021 | BA.1.1 | 95.6 | 120.8 | 461 |
| hCoV-19/Kenya/SME071_MTRH_S27/2021 | BA.1.1 | 95 | 113 | 502 |
| hCoV-19/Kenya/SME072_MTRH_S39/2021 | BA.1.1.1 | 90.6 | 40.7 | 220 |
| hCoV-19/Kenya/SME077_MTRH_S51/2021 | BA.1.1 | 95.7 | 200.9 | 1041 |
| hCoV-19/Kenya/SME078_MTRH_S63/2021 | BA.1.1 | 96.3 | 382.7 | 2277 |
| hCoV-19/Kenya/SME091_MTRH_S75/2021 | BA.1.1 | 98.6 | 404.9 | 1438 |
| hCoV-19/Kenya/SME092_MTRH_S87/2021 | BA.1.1 | 99.8 | 2283.2 | 6175 |
| hCoV-19/Kenya/SME093_MTRH_S4/2021 | BA.1.1 | 98.9 | 613.3 | 2358 |
| hCoV-19/Kenya/SME095_MTRH_S16/2021 | BA.1.1 | 99.8 | 342.3 | 1298 |
| hCoV-19/Kenya/SME099_MTRH_S28/2021 | BA.1.1 | 93.3 | 99.4 | 609 |
| hCoV-19/Kenya/SME103_MTRH_S52/2021 | BA.1.1 | 90.6 | 98.9 | 610 |
| hCoV-19/Kenya/SME106_MTRH_S64/2021 | BA.1.14 | 83.5 | 77.3 | 898 |
| hCoV-19/Kenya/SME108_MTRH_S76/2021 | BA.1.1 | 95.6 | 378.8 | 2055 |
| hCoV-19/Kenya/SME109_MTRH_S88/2021 | BA.1.1 | 99.4 | 854.9 | 2741 |
| hCoV-19/Kenya/SME110_MTRH_S5/2021 | BA.1.1 | 87.8 | 36.6 | 200 |
| hCoV-19/Kenya/SME112_MTRH_S17/2021 | BA.1.1 | 99.7 | 606.6 | 1817 |
| hCoV-19/Kenya/SME116_MTRH_S41/2021 | BA.1.1 | 95.9 | 437.4 | 2126 |
| hCoV-19/Kenya/SME117_MTRH_S53/2021 | BA.1.1 | 97.4 | 122.9 | 395 |
| hCoV-19/Kenya/SME118_MTRH_S65/2021 | BA.1.1 | 91 | 43.8 | 192 |
| hCoV-19/Kenya/SME119_MTRH_S77/2021 | BA.1.1 | 90.7 | 124.2 | 887 |
| hCoV-19/Kenya/SME123_MTRH_S6/2021 | BA.1.1 | 95.8 | 110.2 | 612 |
| hCoV-19/Kenya/SME124_MTRH_S18/2021 | BA.1.1 | 99.2 | 228.4 | 623 |
| hCoV-19/Kenya/SME125_MTRH_S29/2021 | BA.1.1 | 98.2 | 106.6 | 365 |
| hCoV-19/Kenya/SME126_MTRH_S30/2021 | BA.1.1 | 90.1 | 171.6 | 1453 |
| hCoV-19/Kenya/SME127_MTRH_S42/2021 | BA.1.1 | 99.4 | 340 | 1031 |
| hCoV-19/Kenya/SME128_MTRH_S54/2021 | BA.1.1 | 99.2 | 197.4 | 657 |
| hCoV-19/Kenya/SME133_MTRH_S66/2021 | BA.1 | 75.5 | 14 | 90 |
| hCoV-19/Kenya/SME135_MTRH_S78/2021 | BA.1.1 | 92.7 | 110.8 | 807 |
| hCoV-19/Kenya/SME137_MTRH_S90/2021 | BA.1.1 | 93.4 | 89.3 | 482 |
