# Supplementary material for: SARS-COV-2 mutations in North Rift, Kenya
Source: PLoS One. 2025 Jun 6;20(6):e0325133. doi: 10.1371/journal.pone.0325133 (PMC12143566; doi:10.1371/journal.pone.0325133)
Supplement: S2 Table — (DOC) [file pone.0325133.s002.doc]

# Supplementary Table S2

S2 Table: Mutation frequency for each mutated position in Percentage

| **Ref pos** | **Protein** | **Ref var** | **Q var** | **Variant** | **Var name** | **No. of Subjects** | **Mutation frequency in Percentage** |
| --- | --- | --- | --- | --- | --- | --- | --- |
| **241** | 5'UTR | C | T | 241 | 5'UTR:241 | 14 | 31.8 |
| **2470** | NSP2 | C | T | A555A | NSP2:A555A | 8 | 18.2 |
| **3037** | NSP3 | C | T | F106F | NSP3:F106F | 43 | 97.7 |
| **3881** | NSP3 | A | C | I388L | NSP3:I388L | 5 | 11.4 |
| **5386** | NSP3 | T | G | A889A | NSP3:A889A | 42 | 95.5 |
| **6513** | NSP3 | GTT | . | S1265 | NSP3:S1265 | 42 | 95.5 |
| **8393** | NSP3 | G | A | A1892T | NSP3:A1892T | 42 | 95.5 |
| **10029** | NSP4 | C | T | T492I | NSP4:T492I | 43 | 97.7 |
| **11286** | NSP6 | TGTCTGGTT | . | L105 | NSP6:L105 | 42 | 95.5 |
| **11537** | NSP6 | A | G | I189V | NSP6:I189V | 42 | 95.5 |
| **13195** | NSP10 | T | C | V57V | NSP10:V57V | 42 | 95.5 |
| **14408** | NSP12b | C | T | P314L | NSP12b:P314L | 44 | 100.0 |
| **15240** | NSP12b | C | T | N591N | NSP12b:N591N | 5 | 11.4 |
| **16744** | NSP13 | G | A | G170S | NSP13:G170S | 28 | 63.6 |
| **18163** | NSP14 | A | G | I42V | NSP14:I42V | 42 | 95.5 |
| **21762** | S | C | . | A67 | S:A67 | 42 | 95.5 |
| **21764** | S | A | . | A67 | S:A67 | 42 | 95.5 |
| **21767** | S | CATG | . | I68 | S:I68 | 42 | 95.5 |
| **21846** | S | C | T | T95I | S:T95I | 42 | 95.5 |
| **21987** | S | GTGTTTATT | . | G142 | S:G142 | 42 | 95.5 |
| **22193** | S | . | T | I210 | S:I210 | 19 | 43.2 |
| **22195** | S | T | G | N211K | S:N211K | 19 | 43.2 |
| **22197** | S | TA | GC | L212C | S:L212C | 19 | 43.2 |
| **22201** | S | . | AGC | S214 | S:S214 | 19 | 43.2 |
| **22202** | S | . | A | V213 | S:V213 | 19 | 43.2 |
| **22203** | S | . | A | R214 | S:R214 | 19 | 43.2 |
| **22204** | S | T | A | R214R | S:R214R | 19 | 43.2 |
| **22578** | S | G | A | G339D | S:G339D | 37 | 84.1 |
| **22599** | S | G | A | R346K | S:R346K | 36 | 81.8 |
| **22673** | S | TC | CT | S371L | S:S371L | 10 | 22.7 |
| **22679** | S | T | C | S373P | S:S373P | 10 | 22.7 |
| **22686** | S | C | T | S375F | S:S375F | 10 | 22.7 |
| **22813** | S | G | T | K417N | S:K417N | 3 | 6.8 |
| **22882** | S | T | G | N440K | S:N440K | 18 | 40.9 |
| **22898** | S | G | A | G446S | S:G446S | 18 | 40.9 |
| **22992** | S | G | A | S477N | S:S477N | 16 | 36.4 |
| **22995** | S | C | A | T478K | S:T478K | 18 | 40.9 |
| **23013** | S | A | C | E484A | S:E484A | 18 | 40.9 |
| **23040** | S | A | G | Q493R | S:Q493R | 17 | 38.6 |
| **23048** | S | G | A | G496S | S:G496S | 17 | 38.6 |
| **23055** | S | A | G | Q498R | S:Q498R | 17 | 38.6 |
| **23063** | S | A | T | N501Y | S:N501Y | 16 | 36.4 |
| **23075** | S | T | C | Y505H | S:Y505H | 16 | 36.4 |
| **23202** | S | C | A | T547K | S:T547K | 42 | 95.5 |
| **23403** | S | A | G | D614G | S:D614G | 44 | 100.0 |
| **23525** | S | C | T | H655Y | S:H655Y | 42 | 95.5 |
| **23599** | S | T | G | N679K | S:N679K | 42 | 95.5 |
| **23604** | S | C | A | P681H | S:P681H | 42 | 95.5 |
| **23854** | S | C | A | N764K | S:N764K | 38 | 86.4 |
| **23948** | S | G | T | D796Y | S:D796Y | 40 | 90.9 |
| **24130** | S | C | A | N856K | S:N856K | 42 | 95.5 |
| **24424** | S | A | T | Q954H | S:Q954H | 42 | 95.5 |
| **24469** | S | T | A | N969K | S:N969K | 42 | 95.5 |
| **24503** | S | C | T | L981F | S:L981F | 42 | 95.5 |
| **25000** | S | C | T | D1146D | S:D1146D | 42 | 95.5 |
| **25584** | ORF3a | C | T | T64T | ORF3a:T64T | 42 | 95.5 |
| **26270** | E | C | T | T9I | E:T9I | 39 | 88.6 |
| **26577** | M | C | G | Q19E | M:Q19E | 42 | 95.5 |
| **26709** | M | G | A | A63T | M:A63T | 42 | 95.5 |
| **27259** | ORF6 | A | C | M19M | ORF6:M19M | 42 | 95.5 |
| **27807** | ORF7b | C | T | L17L | ORF7b:L17L | 27 | 61.4 |
| **28271** | 3'UTR | A | T | 28271 | 3'UTR:28271 | 42 | 95.5 |
| **28311** | N | C | T | P13L | N:P13L | 42 | 95.5 |
| **28362** | N | GAGAACGCA | . | E31 | N:E31 | 42 | 95.5 |
| **28881** | N | GGG | AAC | RG203KR | N:RG203KR | 42 | 95.5 |
