# Supplementary material for: SARS-COV-2 mutations in North Rift, Kenya
Source: PLoS One. 2025 Jun 6;20(6):e0325133. doi: 10.1371/journal.pone.0325133 (PMC12143566; doi:10.1371/journal.pone.0325133)
Supplement: S1 Fig — (PDF) [file pone.0325133.s003.pdf]

The evolutionary relationships SARS-CoV-2 genomes in North Rift Kenya

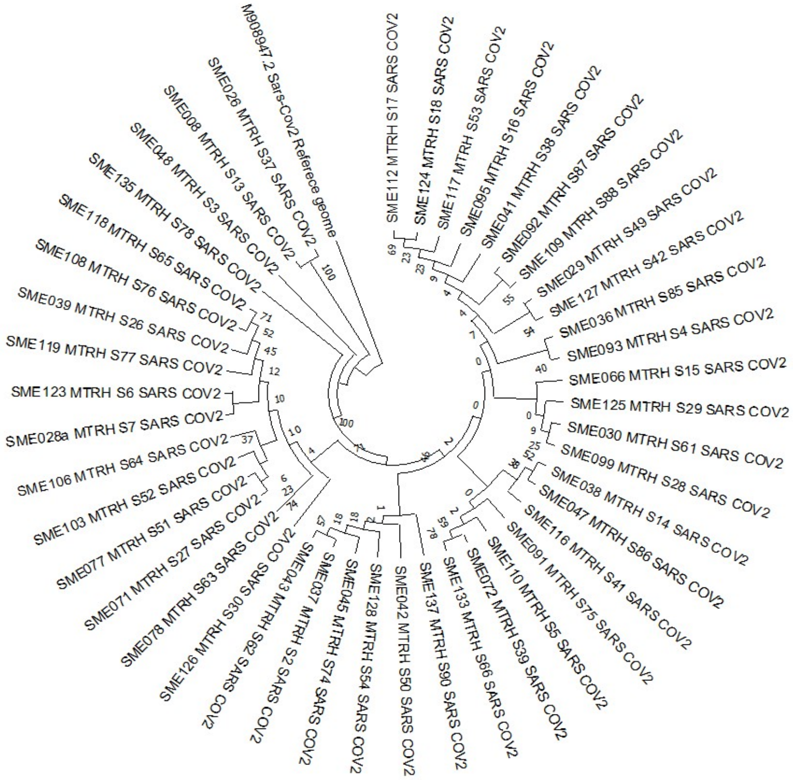

S1 Fig: Evolutionary relationships SARS-CoV-2 genomes in North Rift Kenya

The evolutionary history was determined using the Neighbor-Joining method [1], and the optimal tree is displayed. The percentage of replicate trees in which the associated taxa clustered together in the bootstrap test (500 replicates) are shown next to the branches [2]. Evolutionary distances were estimated using the Maximum Composite Likelihood method [3] and are expressed in terms of base substitutions per site. Site rate variation was modeled with a gamma distribution (shape parameter = 1). The analysis included 45 nucleotide sequences, covering codon positions (1st, 2nd, 3rd, and noncoding). Ambiguous positions were removed for each sequence pair using the pairwise deletion option. The final dataset consisted of 29,888 positions, and all evolutionary analyses were performed with MEGA11 [4].

1. Saitou N, Nei M. The neighbor-joining method: a new method for reconstructing phylogenetic trees. Mol Biol Evol. 1987;4: 406–425. doi:10.1093/oxfordjournals.molbev.a040454
2. Felsenstein J. Confidence Limits on Phylogenies: An Approach Using the Bootstrap. Evolution (N Y). 1985;39: 783. doi:10.2307/2408678
3. Tamura K, Nei M, Kumar S. Prospects for inferring very large phylogenies by using the neighbor-joining method. Proc Natl Acad Sci U S A. 2004;101: 11030–11035. doi:10.1073/pnas.0404206101
4. Tamura K, Stecher G, Kumar S. MEGA11: Molecular Evolutionary Genetics Analysis Version 11. Mol Biol Evol. 2021;38: 3022–3027. doi:10.1093/molbev/msab120
